# Correction: Comparison of professionalism between emergency medicine resident physicians and faculty physicians: A multicenter cross-sectional study

**DOI:** 10.1371/journal.pone.0295894

**Published:** 2023-12-14

**Authors:** Takashi Shiga, Yoshiyuki Nakashima, Yasuhiro Norisue, Tetsunori Ikegami, Takahiro Uechi, Yuhei Otaki, Hidehiko Nakano, Keibun Ryu, Shinjiro Wakai, Hiraku Funakoshi, Shigeki Fujitani, Yasuharu Tokuda

The eighth author’s name is spelled incorrectly. The correct name is: Keibun Liu.
