# Supplementary material for: Comparison of professionalism between emergency medicine resident physicians and faculty physicians: A multicenter cross-sectional study
Source: PLoS One. 2020 Mar 11;15(3):e0230186. doi: 10.1371/journal.pone.0230186 (PMC7065769; doi:10.1371/journal.pone.0230186)
Supplement: S1 File — (DOCX) [file pone.0230186.s001.docx]

Please read the following cases. Recognizing that there may be other approaches, select the single best answer from those listed.

(1) A pharmaceutical company approaches you about a clinical research project involving your office patients. Your patients with high blood pressure will be eligible

to be treated with a new medication that has just been released by the FDA. The object of the study is to evaluate risks and benefits of this medication in an unselected office population. The pharmaceutical company will pay $250 per patient for the expenses generated by the study, and one year’s salary for a data manager and will supply the drug free of charge.

Meetings to discuss the initiation of the study and follow-up results will be held in New Orleans and Honolulu. Your spouse will be invited as the company’s guest to attend these meetings since they will take you away from home.

Participating in the study would be considered appropriate professional behavior if:

A. your patients signed an informed consent;

B. your patients sign an informed consent and your partners approve the study;

C. an oversight committee of the hospital where you have privileges or your regional medical society approves the study;

D. none of the above.

(2) You are practicing hematology and oncology in a suburb of a large metropolitan area. Currently, you refer your patients who require radiotherapy to one or two hospitals in the city depending on where the patients live and the type of problem. A radiotherapist whose knowledge and skill you respect informs you that she will be joining a for-profit national radiotherapy company that is thinking of locating in your area. This new company will bring both the latest equipment and up-graded service to your community. She informs you that an excellent opportunity now exists to invest in this company and that the larger the number of investors from the area, the greater the likelihood the company will locate the unit in your community.

Which of the following statements most accurately assesses the possibility of conflict of interest regarding your investment in this company?

A. An investment will pose a conflict of interest and you should not make it.

B. Your investment will pose no conflict of interest because the new radiotherapy unit will offer superior treatment and will be available to your patients.

C. There is a possibility of conflict of interest that requires that you inform your patients of the investment.

D. Your investment will pose no conflict of interest if you avoid referring your patients to the new radiotherapy unit.

E. There will be no problem of conflict of interest for you if the investment is made by your spouse.

(3) A friend’s 16-year-old daughter visits your office requesting birth control pills. Her family is Catholic and against birth control and premarital sex. She requests you do not discuss this with her parents. After concluding the visit, you return to your desk where you find a message to call the patient’s mother. In the past you have always discussed the daughter’s health and concerns openly. What will you do?

A. Call the mother back and disclose the reason for her daughter’s visit.

B. Return the call and tell the patient’s mother you can’t discuss the matter, knowing this will look suspicious to her.

C. Return the call but be evasive when questioned about the nature of the visit.

D. Don’t return the call.

(4) You are the chief of service at a hospital and a medical student informs you that she smelled alcohol on the breath of an attending physician during morning rounds on more than one occasion. This report is confirmed by another student and a resident. How do you proceed?

A. Approach the physician in question and ask if he/she has a drinking problem.

B. Talk to friend and family members of the physician to see if they suspect a drinking

problem.

C. Review the physician’s file and monitor him/her closely.

D. Report the physician to the Colorado Board of Medical Examiners.

(5) During your rounds with the housestaff team, a male staff member comes up to the group, places his arm around the waist of a female house officer, and thanks her for the terrific job she did she did taking care of one of his patients. You sense that the house officer is made uncomfortable by the gesture.

An appropriate first response would be which of the following?

A. Do nothing, on the basis that the faculty member was simply showing his appreciation for a job well done.

B. Report the incident to the program director as an example of sexual harassment.

C. Tell your colleague, the faculty member, that you thought the gesture was inappropriate and that you were made uncomfortable by it.

D. Ask the resident if the gesture made her uncomfortable.

E. Ask the resident if there are actions she would like you take on her behalf.

(6) An established patient of yours presents with symptoms of depression. This is the second time in three months that the patient has visited you for these complaints.

You wish to start treatment with anti-depressant medication. As you are filling out the prescription, the patient asks you not to document the diagnosis or medication in the chart. She is concerned that her employer will find out about her diagnosis and she

could potentially lose her job like a coworker did.

She knows that her insurance company has access to her diagnosis. How do you proceed?

A. Inform the patient that you must document the diagnosis to provide any treatment.

B. Agree to not document the diagnosis but prescribe the medication anyway.

C. Agree to not document the diagnosis but refuse to provide the prescription.

D. Terminate your relationship with the patient because she is inhibiting your ability to provide adequate care.

E. Document an alternative diagnosis, such as fatigue, and provide the prescription.

The best responses were D for scenario 1, A for 2, B for 3, D for 4, C for 5, and A for 6. The second best responses were C for 1, C for 2, C for 3, A for 4, E for 5, and C for 6.

**Supporting information**

**Barry professionalism Questionnaire used in the present study.**
