# Supplementary figures and images for: Comparison of professionalism between emergency medicine resident physicians and faculty physicians: A multicenter cross-sectional study
Source: PLoS One. 2020 Mar 11;15(3):e0230186. doi: 10.1371/journal.pone.0230186 (PMC7065769; doi:10.1371/journal.pone.0230186)

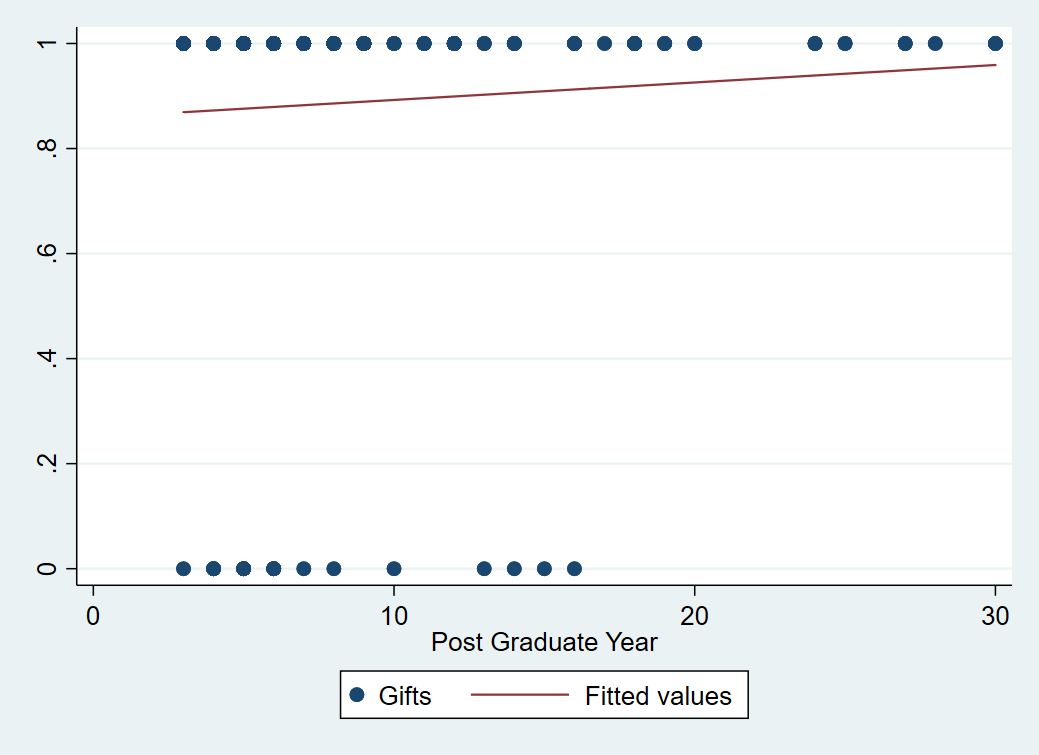

Supplement: S1 Fig — (TIF) [file pone.0230186.s002.tif]

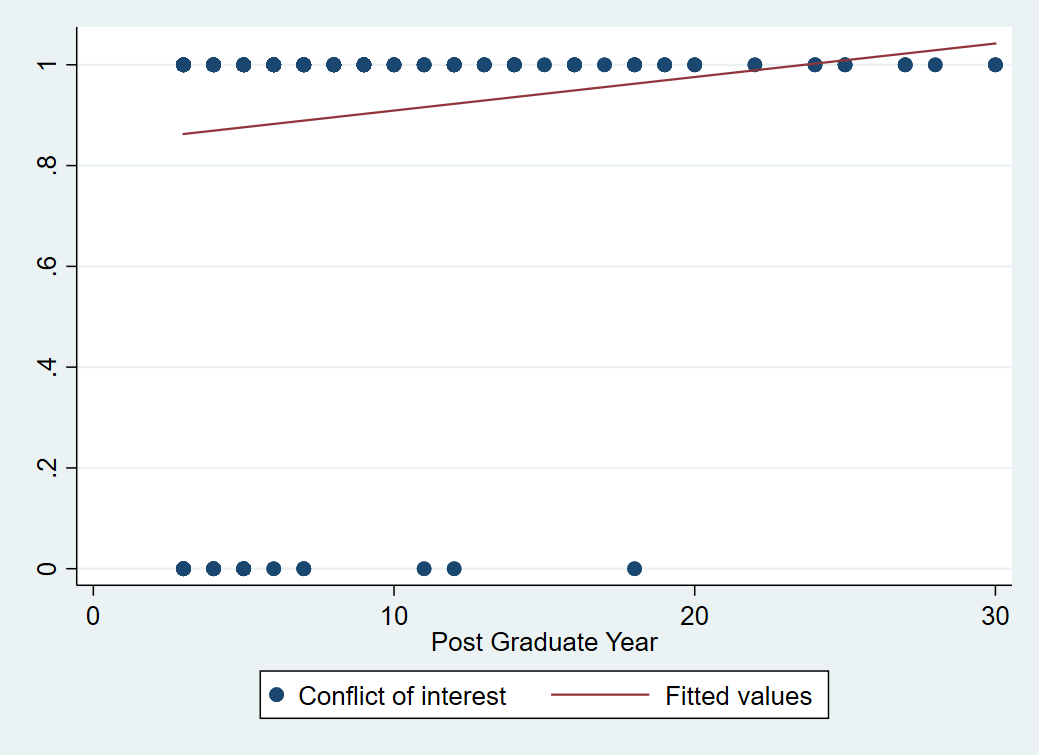

Supplement: S2 Fig — (TIF) [file pone.0230186.s003.tif]

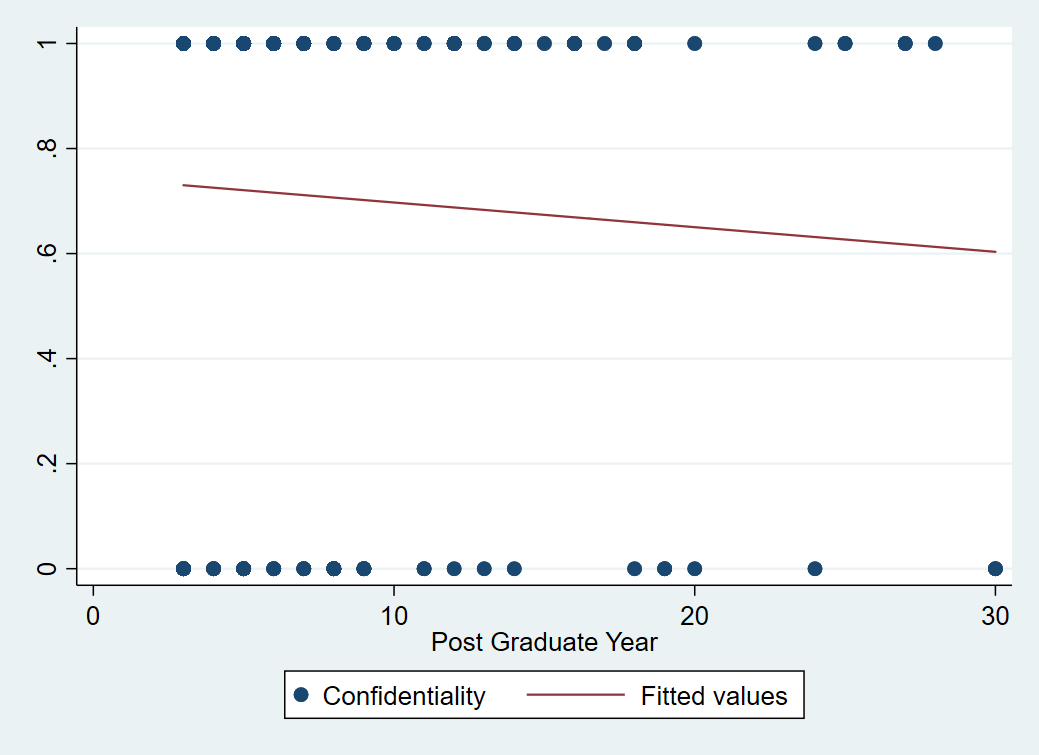

Supplement: S3 Fig — (TIF) [file pone.0230186.s004.tif]

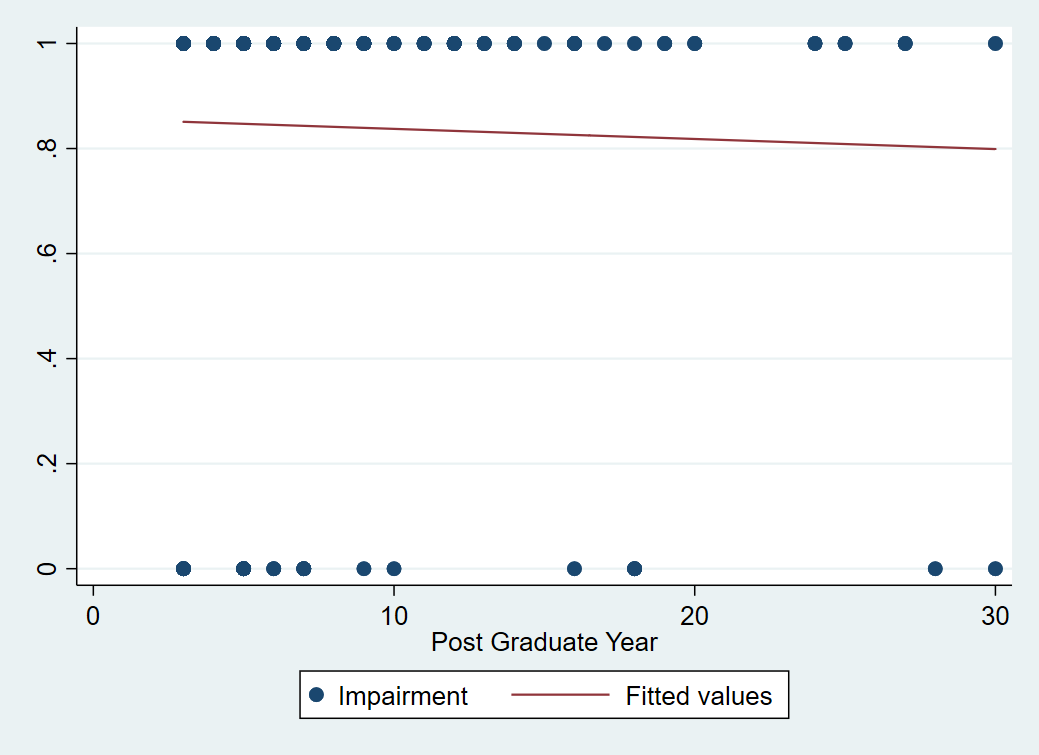

Supplement: S4 Fig — (TIF) [file pone.0230186.s005.tif]

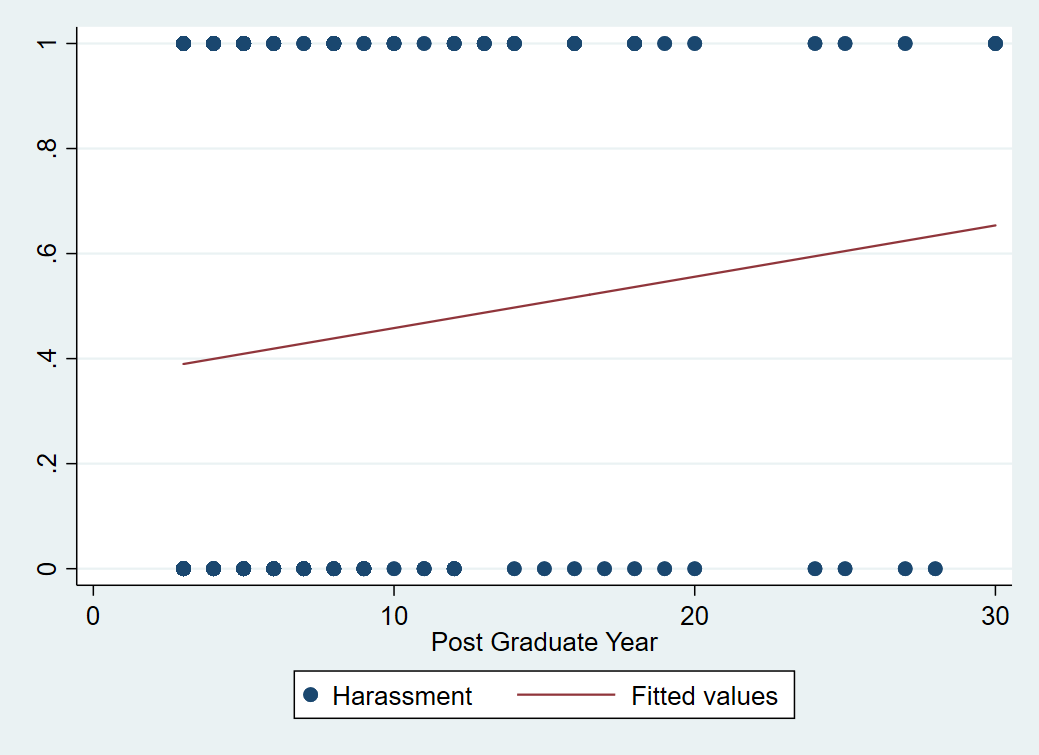

Supplement: S5 Fig — (TIF) [file pone.0230186.s006.tif]

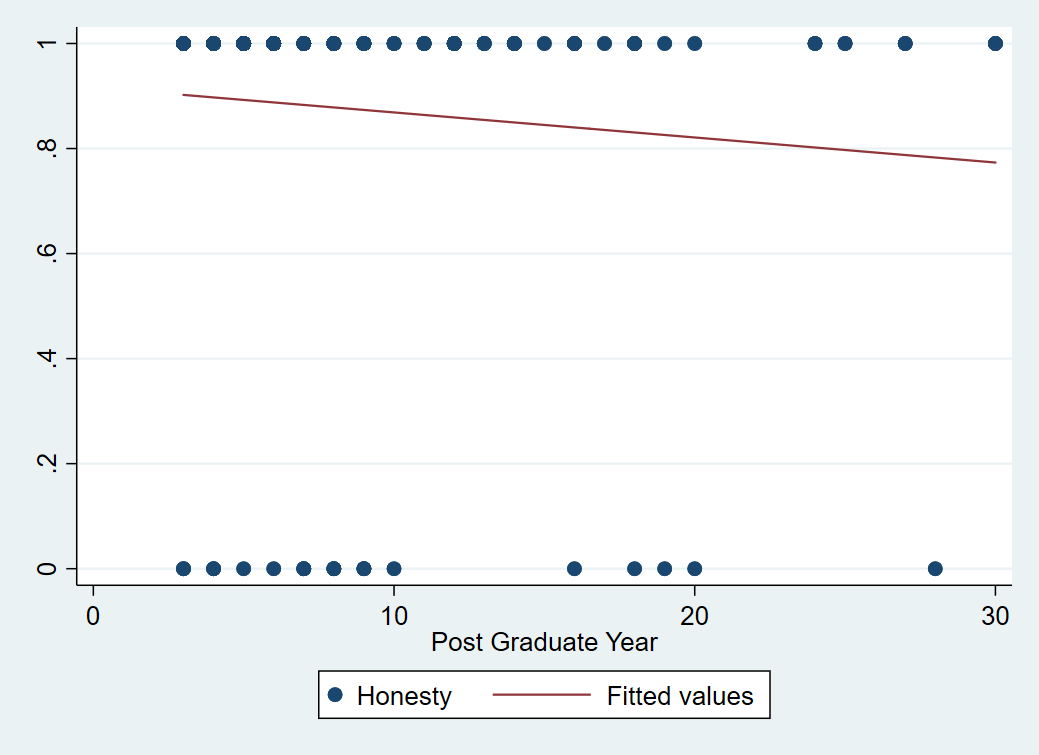

Supplement: S6 Fig — (TIF) [file pone.0230186.s007.tif]

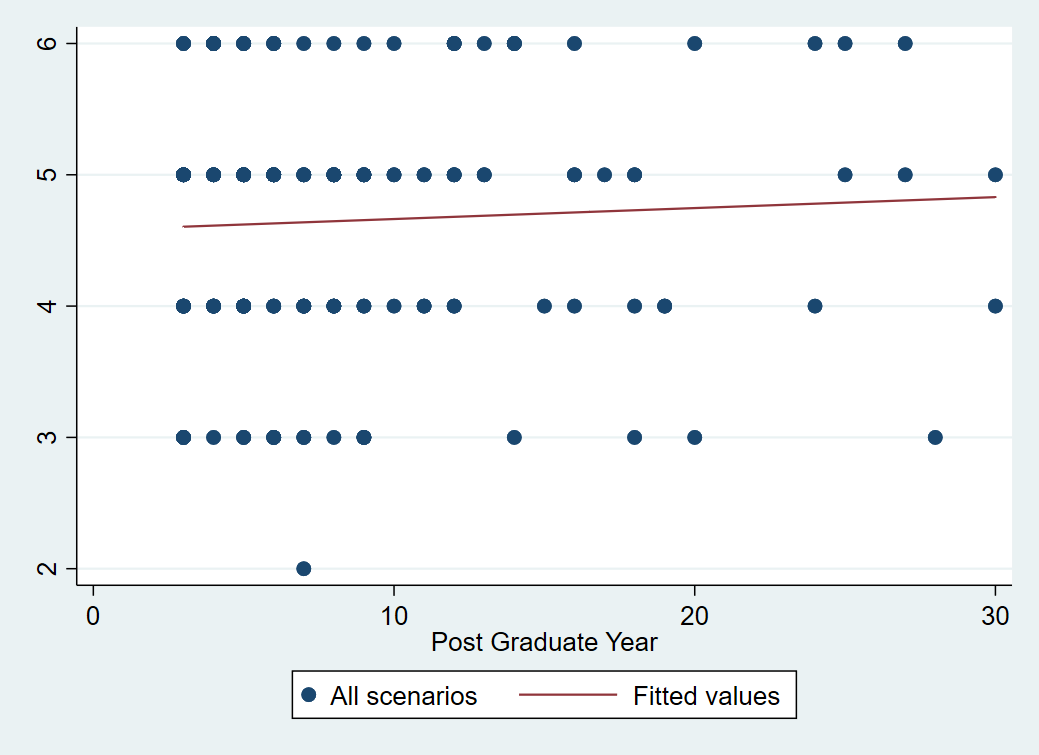

Supplement: S7 Fig — (TIF) [file pone.0230186.s008.tif]
