# Supplementary material for: Comparison of professionalism between emergency medicine resident physicians and faculty physicians: A multicenter cross-sectional study
Source: PLoS One. 2020 Mar 11;15(3):e0230186. doi: 10.1371/journal.pone.0230186 (PMC7065769; doi:10.1371/journal.pone.0230186)
Supplement: Attachment — Submitted filename: Response to Reviewers.docx [file pone.0230186.s009.docx]

**Response to Reviewers**

**Reviewer #2:**

1) **There are still some mistaken expressions to the attitudes towards professionalism as measured by the questionnaire ("medical professionalism is used in line 237, eg).**

***Response:***

We appreciate reviewer’s insightful comments. In the revised manuscript, we tried to use ‘views regarding medical professionalism’ **(Page 17 line 240).**

2) **General English editing is necessary: there is a typing mistake in line 265 ("facial expressions); "focus ON collaborative learning" (line 278).**

***Response:***

We appreciate reviewer’s insightful comments. We have updated as ‘facial expressions’ **(Line 268)** and focus on collaborative learning **(Line 281)** in each section accordingly.

3) **The translation and validation process of the questionnaire cited in Ref 12 did not follow rigorous standards of translation and cultural adaptation as recommended in various translation guidelines. Authors should point it as a limitation to the study design.**

***Response:***

We appreciate reviewer’s insightful comments. We have updated this point at the limitation section **(Page21 line 307).**

**Journal requirement:**

**While revising your submission, please upload your figure files to the Preflight Analysis and Conversion Engine (PACE) digital diagnostic tool, https://pacev2.apexcovantage.com/. PACE helps ensure that figures meet PLOS requirements.**

***Response:***

We appreciate journal officer’s instructions. We have checked our images through PACE system and uploaded to the submission system accordingly.
